# Medical Society of the County of Albany

**Published:** 1874-07

**Authors:** F. C. Curtis

**Affiliations:** Secretary


ART. III.—*Medical Society of the County of Albany. Semi-Monthly Meeting, held April 6th*, 1874. Reported by F. C. Curtis, M. D., Secretary.

The Society met at the usual place and hour.

In the absence of the President, Dr. W. H. Craig, was elected Chairman *pro tem*, and called the meeting to order.

After the minutes of the last meeting had been read and approved,

Dr. Devol presented a case of intra-cranial abscess resulting from inflammation of the internal ear. The patient had been subject to attacks of earache for a number of years, otherwise his health had been good, and in the intervals of the attacks he suffered little inconvenience. Ten days before death, a very severe attack came on. Pain was excruciating, demanding active treatment for its relief. Four days before he died, violent rigors set in. Coma soon followed and continued till death.

An autopsy was made with the following result: The left hemisphere of the brain was conjested throughout, the lateral ventricles being distended with clear serum. The surface of the right hemisphere was covered with pus, in quantity sufficient to compress it considerably. The inflammation did not extend into the sulci much, and the base of the brain was not involved. The gray matter in section was of a greenish brown tinge. On the right side, between the dura mater and the petrous portion of the temporal bone, was a collection of pus to the amount of nearly a fluid ounce, limited within a space two inches in diameter. There was no perforation of the dura mater (which was exhibited). This abscess communicated with the internal ear which was filled with pus. The tympanum was destroyed and a probe passed freely into the internal ear from the external meatus.

The thoracic organs varied little from normal. So.also the abdominal, excepting there was marked conjestion of the kidneys.

Dr. Stonehouse quoted from a recent author the opinion that there is a preference for the right side in abscess following otorrhœa, and asked the experience of the Society in the matter; also whether it ever followed acute otitis.

Dr. Davis narrated two cases, in both of which the left ear was affected. One of these began with earache, there being some swelling and tenderness back of the ear. Suppuration took place and the earache ceased, but the region back of the ear continued swollen and tender. Suppuration continued for weeks, and a large piece of the temporal bone became detached and moveable, pus welling up when it was pressed upon. The bone never came away. After three or four months, during which the general condition of the patient continued good, symptoms of compression of the brain set in, and he became comatose and died. No autopsy was made. The second case began with moderate earache, followed by suppuration. Abscess formed and discharged, after a year and a half, pieces of bone. Recovery took place only after the lapse of two years. Hearing was not affected.

Dr. Devol spoke on the subject of burns, alluding to a case of extensive and fatal burning of the body, of which he had recently had charge. He enquired how large an extent of surface burned was necessarily fatal?

Dr. James S. Bailey said that his experience had been somewhat extensive in burns of the body. He had met with many cases at the South, where it was very common for negro women to set fire to their clothing while burning brush, in clearing land. He was of the opinion that burning one-eight of the surface, especially if it be the chest and abdomen, is fatal. He detailed one case where death occurred in six hours from shock, and another in which the patient died in three months from exhaustion.

The question was raised as to the cause of death in burns by Dr. Devol. He had noticed that patients extremely burned, as for instance from falling into a vat of hot liquid, complain, when conscious sufficiently to express themselves, of feeling very cold.

Dr. Van Derveer attributed this feeling to nervous shock. He had once amputated a limb without chloroform, and the patient told him that he only felt the cutting of the skin. The skin is very rich in nerves. Holt's dilator of the urethra has in like manner caused fatal shock. As to the treatment of burns he liked nothing better than the mixture of olive oil and lime water Skin grafting comes in as an after treatment where applicable. He had seen recovery when more than one-eighth of the surface was burned.

Dr. Lewis Balch mentioned several cases of burns which he saw during his service in the New York and Brooklyn City Hospitals. One was burned from the knees down by falling into a vat. During the shock following, stimulants were given. The limbs were dressed with carron oil, the granulations being occasionally touched with nitrate of silver. In another case the surface was painted during granulation with a solution of nitrate of silver, the pains from which was intense, but the result favorable.

Dr. Curtis said that in a case of extensive burn of the thigh, in the General Hospital at Vienna, in the wards of Professor Hebra, he had seen Lister's bandage used, which consisted of a paste containing carbolic acid spread on tin or lead foil. This was kept constantly applied. The wound granulated evenly under it, portions being painted occasionally with a solution of nitrate of silver. In the case of a ballet dancer, whose body was so extensively burned that no hope of recovery was entertained, great relief was afforded by immersing the entire body to the neck under water, by means of a bath tub arranged with a float to support the body. The bath had been arranged for the treatment of certain cases of skin diseases, such as pemphigus, patient having been kept for weeks constantly in it.

Dr. Devol gave the following formula for use in burns: Glycerine, yolk of egg and butter of cacao of each equal parts. This forms an effectual defense. In fatally extensive burning, however, the body should be covered with olive oil by means of cloths saturated in it, this being ready at hand, and not waiting for more complicated remedies.

Dr. T. D. Crothers read a paper on "The Treatment of Inebriation," of which the following is a synopsis:—

After giving a review of the literature and unsettled theories concerning the nature and pathology of inebriates, the doctor mentioned the following summary of facts, which are confirmed by the experience and observation of medical men.

1st. Inebriety is a physical disease, often hereditary, and its most active cause is alcohol.

2d. This disease in all cases affects certain brain districts, and the nutritive functions which it controls, and may begin indirectly from powerful mental emotions, previous disease or injury.

3d. Inebriety, from all causes, results in organic lesions of both the physical and nervous system, which make it the most formidable and complicated disease known.

4th. The experience of fifteen years has shown that this disease can be cured as other diseases are. That its natural tendency is towards recovery, and a large proportion of recent cases may be completely restored.

The doctor affirmed that this disease presented such various symptoms in each case, that its successful treatment depended upon the particular knowledge of the patient, his history and his habits. He also discussed the general and special symptoms of inebriety, and their pathological meanings, observing that the general symptoms always indicated impaired constitutional power, and debility of organic life, whether apparent or not to the senses. The observation of all writers on this subject agree that the inebriate has a debilitated physical system, which is imperfectly nourished, because of the poisonous alcohol taken into the stomach, interfering with the process of repair. The special symptoms of inebriety point to molecular disturbance of the nutrient nerve centres, functional paralysis of the stomach with general diminution of the co-ordinating nerve force. This may be rapidly followed by organic changes in the stomach, liver, kidneys, and lastly of the heart and lungs. The organic changes in the brain begin after the lesions of other organs. But the functional changes of the brain are first in order of time.

The doctor continued: All inebriates have a defective constitution and weakened will power. The function of repair, by which the body is built up and sustained, it deranged, and the mental or nervous system must suffer. The first indication in treatment is to restore the general health. To do this, food is of the first importance. It was the boast of the celebrated chemist Liebig, "that, given the quality, quantity and regularity of food, he could determine the character and enterprise of any race." The hint we are taught by this is that food is a prominent agent in forming the mental and physical health of the individual or nation. A carefully regulated diet, adapted to meet the wants of each case, has been found the most valuable in treatment. Add to this such tonics as act locally on the stomach and nervous system, including such remedies as iron, quinine, bismuth, pepsin, etc. Bathing and regularity of sleep, well ventilated rooms and sunlight are indispensable. Of sunlight we begin to learn that it is a tonic, without which we can not secure health and vigor. By these general means the health of the inebriate is improved, and if we accompany them with cheering mental associations and removal from all exciting causes, complete restoration will follow in many cases.

The mind is treated by rest and change of thought, and companions who have like motives and hopes. * * * Good food and excellent care build up the physical, change of thought, occupation and surroundings, restore the mental, and this constitutes the general plan of treatment.

In answer to the question what can be done scientifically for this disease, Dr. Crothers remarked that for half a century and more, insanity has been treated and studied practically. Large wealthy hospitals, with men of genius and talent to manage them exist all over the country; ponderous volumes of discoveries and speculations on the pathology and treatment of insanity fill the medical libraries. The study and literature have grown with the science until it has become a separate department in the practice of medicine. Statistics of the best hospitals indicate that from 35 to 40 per cent. of all the patients go away cured. Inebriety began to attract attention as a disease fifteen years ago, and since then has been partially recognized by the profession and very imperfectly studied. At present eight asylums exist in the country, many of these but recently started, and all more or less lacking experience and practical adaptation; and yet the statistics of cured exceed those of the best appointed insane asylums. Over 40 per cent. go away entirely recovered, and remain so as far as the observation of three years has determined. Here is a hint of the possibilities of science in behalf of the poor inebriate that we cannot neglect. If with our present imperfect knowledge and means of treatment we are able to cure over 40 per cent. of this class, what may not be done in the progress of the future? If we should never be able to cure more than 40 per cent. and restore that number back to usefulness and society, the value of the work would be inestimable. Hospitals and asylums for this special work bring isolation from temptation and exciting causes, also change of thought and habits with regularity and uniformity of living. Add to this the excellent facilities to carry on judicious medical and hygenic treatment, etc. The value of treatment statistically is significant when we remember that of the six hundred thousand inebriates in this country, at least four hundred thousand would, if in health, be producers. The average income of this class at a low estimate would be for each five hundred dollars a year. At present they without doubt absorb this amount, making the enormous sum of two hundred millions of dollars per year lost to keep up this class, which should be producers of this amount. This is a low estimate considering the varied surroundings of each inebriate, with its connections, which to a greater or less extent become producers or absorbers with him. Not less than one billion of dollars is lost yearly in the country by this class. If the present comparatively imperfect means of treatment prove that 40 per cent. can be restored to health again, the value of treatment is a financial problem of unbounded interest.

The efforts of philanthropists, through the means of societies, appealing to the moral and mental faculties of the inebriate, are not successful, because they ignore the physical system, and attempt to build up a weakened will power which depends entirely upon the constitution. We can only reach the mental and moral through the physical, and the efforts to pledge the poor inebriate, whose debilitated system will fail to sustain his mental desires, is worse than useless. The pledge repeatedly broken, from causes beyond control, hastens the final ruin of the inebriate by lowering all self-respect and mental conscience. Both practically and scientifically, the present mode of reaching this malady by appeals, associations and agitations, is a sad reflection on the intelligence of the age. The legal method of treatment by fines and imprisonment, and the old medical treatment which never recognized inebriety as a disease until it reached the last stage, that of delirium tremens, are degrees of ignorance which will be regarded with amazement by the future. Dr. Willard Parker of New York, in a private letter, writes: "The profession needs light as much as the laity. We must grapple with this subject, and understand it, then by our united efforts we can do more than all the temperance movements of the age." The temperance labors of to-day only benefit the inebriate by calling attention to his condition, and the real reform sought for must come from the medical profession, and their studies of this malady in asylums and hospitals. This is confirmed by statistics which indicate that inebriety is increasing despite all popular movements at reform. Scientific inquiry has been turned towards this subject, and we are just beginning to recognize and understand the true methods of reaching and preventing this disease. The success of the Binghamton asylum and its recognition by the State as one of its great charities are significant of the future. The efforts made in several States to organize similar institutions show that public opinion is moving in the right direction at last. No field of practical medicine promises such widespread and lasting results to both individual and State. Inebriety and its management are great facts yet in their infancy, which at no distant day will be studied in the asylum in every city and county in the country.

The present temperance agitation is an opportune moment for the medical profession to come forward as teachers and educators of public opinion, and in this way attempt to guide this lavish outpouring of energy and wealth of purpose, that would convert rumsellers and save inebriates by the thousands. This tide of public opinion should be turned into the channels of practical science, stimulating the birth and growth of inebriate asylums and opening wide the doors of its health fountains that the diseased inebriate may drink and live again. Teach men that inebriety is a dangerous disease, and the one hundred thousand victims yearly will dimish to an insignificant number, and rumselling will also die out as the malaria of western river bottoms.

An observation by Dr. Bowditch, of Boston, was spoken of—that north of an isothermal line of an average temperature of 50° drunkards commit crime when intoxicated, while south of it they are harmless, presenting milder types of mental derangement.

Dr. Van Deveer enquired what were the changes in the brain of inebriates; whether it lay in a destruction of the phosphatic fats, of nerve fibre, nerve cells or what. Why should this change spoken of by Dr. Crothers be the primary lesion, instead of these of the stomach, liver or other organs, and when men stop drinking at once, are these brain conditions removed?

He also spoke of the hypodermic use of alcohol, as recently suggested, in shock of extreme depression.

DR. CROTHERS explained the change in the brain as one of function rather than of structure.

Remarks were made by DR. DAVIS and others on the subject.

